# Glycerol-Based Cryopreservation of CELT-Fat: Identification of the Optimal Concentration in a GMP-Compatible Protocol

**DOI:** 10.3390/cells15070605

**Published:** 2026-03-28

**Authors:** Lukas Prantl, Oliver Felthaus, Andreas Eigenberger, Dmytro Oliinyk, Tom Schimanski

**Affiliations:** 1 Department of Plastic, Hand and Reconstructive Surgery, University Hospital Regensburg, Franz-Josef-Strauss-Allee 11, 93053 Regensburg, Germany; lukas.prantl@ukr.de (L.P.); oliver.felthaus@ukr.de (O.F.); dmytro.oliinik@ukr.de (D.O.); 2 Medical Device Lab, Regensburg Center of Biomedical Engineering (RCBE), Faculty of Mechanical Engineering, Ostbayerische Technische Hochschule Regensburg, Galgenbergstraße 30, 93053 Regensburg, Germany

**Keywords:** adipose tissue, lipoaspirate, CELT-Fat, cryopreservation, glycerol, autologous fat grafting, GMP compatible

## Abstract

Background: Autologous fat grafting is widely used in reconstructive, aesthetic and regenerative surgery and often requires repeated applications. Cryopreservation of lipoaspirate enables autologous fat banking and off-the-shelf availability; however, its clinical implementation is limited by freezing-induced tissue injury, regulatory requirements and uncertainties regarding the optimal preservation protocol. Glycerol is a biocompatible cryoprotective agent with promising preliminary data. Nevertheless, the optimal concentration for lipoaspirate cryopreservation remains unknown. The aim of this study was to determine the optimal glycerol concentration for preservation of adipose tissue processed according to the Cell-Enriched Lipotransfer (CELT) protocol in clinically relevant volumes under GMP-compatible conditions. Methods: Lipoaspirates from 10 patients were processed by centrifugation according to the CELT protocol and allocated into experimental groups: fresh unfrozen control, frozen samples without cryoprotectant, frozen samples with PBS, and frozen samples supplemented with glycerol in concentrations ranging from 10% to 60%. Samples were cryopreserved using a controlled freezing rate at a temperature of −80 °C for 24 h. Large-volume cryopreservation was additionally performed with the best concentration of glycerol. Post-thaw tissue quality was assessed by resazurin assay of whole tissue, stromal vascular fraction (SVF) cell live/dead counting, and resazurin assay after short-term cell culture. Results: Glycerol supplementation improved post-thaw tissue viability compared with cryopreservation without cryoprotectant or with PBS alone. An optimal concentration range between 10% and 30% glycerol was identified, with highest preservation of metabolic activity and surviving cell yield observed at 20%. Higher glycerol concentrations resulted in a marked decline in tissue quality. Cryopreservation in large volume was feasible and did not impair post-thaw viability compared with small-volume samples. Conclusions: Glycerol-based cryopreservation allows effective and GMP-compatible preservation of human lipoaspirate. An optimal glycerol concentration range was identified, enabling large-volume fat banking without compromising tissue quality. This protocol provides a clinically applicable strategy for autologous fat storage and may facilitate repeated reconstructive and regenerative treatments.

## 1. Introduction

Autologous fat grafting has become an integral component of reconstructive and aesthetic surgery [1,2,3]. Beyond its volumetric effect, adipose tissue represents a rich source of stromal vascular fraction cells which contribute to tissue regeneration and neovasculogenesis [4,5,6,7,8]. These biological properties have expanded the clinical spectrum of fat grafting from purely aesthetic procedures to regenerative indications such as chronic wound treatment, joint disorders and soft tissue augmentation after oncologic surgery [9,10,11,12,13,14,15,16].

In many scenarios, repeated fat injections are required to achieve or maintain stable clinical results [17,18,19]. This is particularly relevant in patients after cancer therapy upon reconstruction, with chronic wounds, or paraplegic patients at risk for pressure ulcer formation. Repeated liposuction procedures are associated with surgical and anesthesiologic risk. The concept of fat biobanking, defined as the long-term storage of autologous lipoaspirate for repeated future applications, therefore represents an attractive strategy to decrease patient morbidity and harvesting-associated side effects [20].

Good manufacturing practice (GMP) compatible cryopreservation of adipose tissue, however, remains technically challenging. In addition to considerations regarding sterile work processes, mature adipocytes are susceptible to freezing injury due to their fragile cell membranes and limited tolerance of osmotic stress [21,22]. Ice crystal formation, osmotic stress and cryoprotectant toxicity contribute to cell damage, impaired graft viability and unpredictable clinical outcomes [23,24,25]. In addition, any protocol intended for clinical use must fulfill strict requirements, including sterility, traceability, validation and compatibility with good manufacturing practice standards.

Dimethyl sulfoxide (DMSO) has been widely used as a cryoprotective agent in cell therapy and adipose tissue preservation [23,26,27,28]. Although effective in reducing cryopreservation-associated cell damage, DMSO is associated with cytotoxicity, inflammatory reactions and potential systemic adverse effects [24,29,30]. These concerns are particularly relevant in aesthetic and reconstructive surgery, since in aesthetic procedures even minimal side effects are hardly acceptable in the absence of a medical indication, whereas in reconstructive surgery patients are often severely ill, making them especially vulnerable to additional risks. The introduction of potentially toxic substances into such clinical settings raises safety concerns.

Glycerol represents an alternative cryoprotective agent with a long history of clinical use in cryobiology and blood banking [31,32].

Preliminary experimental and clinical studies have demonstrated promising results for glycerol-based cryopreservation of adipose tissue, suggesting preserved tissue architecture, cell viability and regenerative potential [33,34]. However, the optimal glycerol concentration remains unclear, and no standardized, GMP-compatible protocol for large-volume clinical fat banking is currently established.

In addition to the choice of cryoprotectant, the cryopreservation bag and handling procedure play a crucial role in ensuring sterility. Most experimental studies rely on small sample volumes in laboratory vials, which are not suitable for clinical fat storage. The availability of sterile, validated cryopreservation bags for lipoaspirate offers the possibility to integrate fat banking directly into the operative setting, minimizing contamination risk and enabling closed system processing. The present study was designed to validate a GMP-compatible cryopreservation protocol for human lipoaspirate using glycerol as cryoprotective agent. The adipose tissue was processed according to our established Cell-Enriched Lipotransfer (CELT) protocol, which has demonstrated consistent clinical efficacy and safety. Processing according to the CELT protocol is also of particular relevance for subsequent cryopreservation, as it enables the near-complete removal of residual tumescent solution, blood components, and the oil fraction derived from disrupted adipocytes. Importantly, this purification is achieved using optimized centrifugation parameters that avoid excessive shear stress and do not compromise cellular integrity or the viability of the stromal vascular niche. The CELT method is specifically designed to preserve the structural architecture of adipose tissue while maintaining high viability of adipose-derived stromal/stem cells (ASCs), pericytes, endothelial progenitor cells, and other regenerative cell populations within the stromal vascular fraction (SVF). Furthermore, the optimized removal of free lipids and aqueous components prior to freezing may enhance cryostability by reducing ice crystal formation, osmotic stress, and post-thaw inflammatory responses. This may ultimately contribute to improved graft retention, structural stability, and regenerative potential following thawing and transplantation.

We systematically evaluated different glycerol concentrations, ranging from 10 to 60 percent, and compared their effects on the resazurin assay results of whole tissue, stromal vascular fraction cell live/dead ratio, and resazurin assay after short-term cell culture.

After that, we assessed the feasibility of cryopreserving large volumes of adipose tissue in a large quantity of 100 mL and investigated the influence on post-thaw quality.

We hypothesized that glycerol-based cryopreservation can provide a safe and effective alternative to DMSO and that large-volume fat banking is feasible without compromising tissue quality. Furthermore, we explored qualitative changes observed after thawing, including oil release.

By establishing a clinically applicable cryopreservation protocol, this study aims to contribute to the development of off-the-shelf autologous fat grafting strategies.

## 2. Material and Methods

### 2.1. Harvesting and Preparation of Lipoaspirate

Lipoaspirate samples were obtained from 10 patients (8 female and 2 male) with a mean age of 42.3 ± 10.0 years and a mean body mass index of 28.3 ± 3.3 kg/m^2^. All patients provided written consent prior to the tissue collection. The study protocol was approved by the Ethics Committee of the University Hospital of Regensburg (24-3640-101).

Liposuction was performed according to a standardized tumescent technique. A solution containing 0.9% sodium chloride supplemented with epinephrine (1:200,000) was infiltrated into the donor site using a 2.5 mm infiltration cannula and allowing exposure for 15 min. Fat harvesting was performed using a 3.8 mm aspiration cannula connected to a water-assisted liposuction system (Body-Jet^®^, Human Med AG, Schwerin, Germany) applying a constant negative pressure below 0.5 mbar.

After sedimentation, the aspirates were processed by centrifugation to remove excess tumescent fluid and free oil via the CELT protocol [35,36,37]. Briefly, lipoaspirate was transferred into 10 mL Luer-Lock syringes (Becton Dickinson, Madrid, Spain) and centrifuged at 1600 rcf for 2 min (Rotina 380R, Andreas Hettich GmbH & Co. KG, Tuttlingen, Germany). The infranatant aqueous phase and the supernatant lipid layer were discarded, and the remaining tissue fraction, corresponding to the processed CELT fat, was obtained in typical laboratory-scale quantities for all downstream in vitro experiments. This was done to generate a standardized adipose tissue fraction with minimal residual fluid content to ensure comparability across experimental conditions.

### 2.2. Preparation of Cryoprotective Solutions

Glycerol (Sigma Aldrich, St. Louis, MO, USA) was used as cryoprotective agent and diluted with phosphate-buffered saline (PBS) to prepare solutions with glycerol concentrations of 0, 10, 20, 30, 40, 50 and 60% (*v*/*v*). The glycerol concentrations indicated in Table 1 refer to the initial cryopreservation solutions and are reduced by half upon mixing with CELT fat at a 1:1 ratio, resulting in the final concentrations present in the frozen product.

The following experimental groups were defined:

**Table 1:** All experimental groups and how they are referred to in this study.

Control	Fresh Unfrozen Control CELT-Fat
*w*/*o*	Frozen without CPA
PBS	100% phosphate-buffered saline
glycerol 10%	Glycerol mixed with PBS 10/90% (*v*/*v*)
glycerol 20%	Glycerol mixed with PBS 20/80% (*v*/*v*)
glycerol 30%	Glycerol mixed with PBS 30/70% (*v*/*v*)
glycerol 40%	Glycerol mixed with PBS 40/60% (*v*/*v*)
glycerol 50%	Glycerol mixed with PBS 50/50% (*v*/*v*)
glycerol 60%	Glycerol mixed with PBS 60/40% (*v*/*v*)

Samples were frozen for 24 h, −80 °C.

### 2.3. Freezing and Thawing Protocol

The fresh control group was analyzed right after preparation. All samples designated for cryopreservation were transferred into cryogenic containers and placed in controlled-rate freezing boxes (Nalgene™ Cryo 1 °C Freezing Container, Thermo Fisher Scientific, Waltham, MA, USA) to achieve a cooling rate of approximately 1–2 °C per minute.

Thawing was performed in a 37 °C water bath for 3.5 min giving the samples enough time to fully thaw.

### 2.4. Cryopreservation of Large Volumes

In the experiments on cryopreservation of large volumes, we cryopreserved 100 mL of CELT fat and compared them to standard laboratory-scale samples of 0.7 mL. Both volume groups were supplemented with glycerol as a cryoprotective agent in a concentration of 20% (*v*/*v*) in a 1:1 ratio. In addition, a control group without any cryoprotective agent was included (*w*/*o*), consistent with the experimental design of the preceding experiments. For comparability across conditions, equal volumes required for each respective assay were taken from both the 0.7 mL 20% glycerol and the 100 mL 20% glycerol samples, and all downstream analyses were performed using identical assay volumes of 50 µL and 0.7 mL. As the control group, the unfrozen samples from the same patient were used.

For evaluation of large-volume cryopreservation under clinically applicable conditions, lipoaspirate samples were transferred into bags feasible for up to 100 mL. After mixing with equal amounts of glycerol PBS mixture of 20/80% (*v*/*v*), the bags were sealed and cryopreserved in a polypropylene box filled with 100% isopropanol for a slow controlled freezing of 1–2 °C at −80 °C.

Thawing was performed in a 37 °C water bath until complete thawing of the tissue content was achieved after approximately 10 min. Samples of the fat tissue were evaluated in comparison to samples that were frozen in smaller volumes. Before thawing, a resazurin assay of whole tissue, stromal vascular fraction cell live/dead counting, and resazurin assay after short-term cell culture were performed, as well as a measurement of free lipids after thawing.

### 2.5. Vitality Assay of Whole Tissue

Tissue viability was assessed using a resazurin assay. For all viability assays, a standardized volume of 50 µL per sample was used, and fluorescence values were normalized to the respective non-cryopreserved control from the same patient to ensure comparability across different sample sizes and conditions. Aliquots of 50 µL CELT fat were incubated in quadruplicates with 45 µL of 0.7 M resazurin (Sigma Aldrich, St. Louis, MO, USA) and 405 µL of α-MEM supplemented with fetal calf serum (FCS).

Samples were incubated for 1 h at 37 °C under constant rotation (10 rpm). Afterwards, all samples were centrifuged at 525 rcf for 2 min, and 100 µL of the aqueous phase from each sample was transferred into a 96-well plate. Fluorescence intensity was measured using a plate reader (VarioScan, Thermo Scientific, Waltham, MA, USA) at an excitation wavelength of 560 nm and an emission wavelength of 590 nm. Fluorescence intensity values were used as an indirect measure of metabolic activity and therefore cell viability.

### 2.6. Live/Dead Ratio of Stromal Vascular Fraction Cells

For isolation of stromal vascular fraction (SVF) cells, 0.7 mL of each tissue sample was digested with an equal volume of 0.2% collagenase type I (Sigma Aldrich, St. Louis, MO, USA) and incubated for 60 min at 37 °C under constant rotation with 10 rounds per minute. The digestion mixture was filtered through 250 µm tissue strainers (Thermo Fisher Scientific, Waltham, MA, USA) and centrifuged at 500 rcf for 2 min to remove undigested material and bigger particles.

The enzymatic reaction was stopped by adding twice the volume of α-MEM containing 10% fetal calf serum. Samples were centrifuged at 700 rcf for 7 min and the resulting cell pellets were resuspended in 700 µL PBS. For cell counting, 50 µL of the suspension was mixed with an equal volume of 0.4% trypan blue, and cells were counted in a Neubauer counting chamber at 40× magnification under consideration of live and dead cells. The number of cells counted in the defined grid areas was multiplied by the chamber specific factor of ×1000 and adjusted for the dilution factor introduced by trypan blue staining (1:1), resulting in the total cell number per mL of suspension. The live/dead ratio was calculated as standard viability, defined as the number of living cells divided by the total number of cells (living cells + dead cells). In the following, this parameter is referred to as the live/dead ratio. The results were normalized to the control group from each corresponding patient for better comparison and to eliminate potential intraindividual differences.

### 2.7. Vitality Assay in a Cell Culture

To assess the functional viability of isolated cells, 200 µL of each cell suspension obtained for the live/dead ratio and described in the subsection above was seeded into wells of a 24-well plate and cultured for 24 h in α-MEM supplemented with 10% FCS and 1% penicillin/streptomycin at 37 °C and 5% CO_2_. After washing twice with PBS, wells were incubated with 500 µL of a resazurin mixture with culture medium for 2 h. Fluorescence intensity was measured as described above. This assay was designed to assess the functional survival of cells derived from 0.7 mL of CELT fat of various treatments, i.e., their ability to attach to the culture surface, survive, and maintain metabolic activity over 24 h. Fluorescence intensity was normalized to the unfrozen control to allow comparability across experiments.

### 2.8. Statistical Analysis

All quantitative data were normalized to the corresponding fresh control samples and expressed as mean ± standard deviation. Group comparisons were performed using paired Student’s *t*-tests. The comparison of interest from a biologically driven standpoint was the best performing condition versus the next-best performing condition. Other conditions are shown for reference, and no Student’s *t*-test was performed for these groups. Normal distribution was verified using the Kolmogorov–Smirnov test. A *p*-value < 0.05 was considered statistically significant. Effect sizes were calculated using Cohen’s d. Descriptive statistics were applied for datasets with limited replication.

## 3. Results

### 3.1. Vitality Assay of Whole Tissue

**Figure 1:**
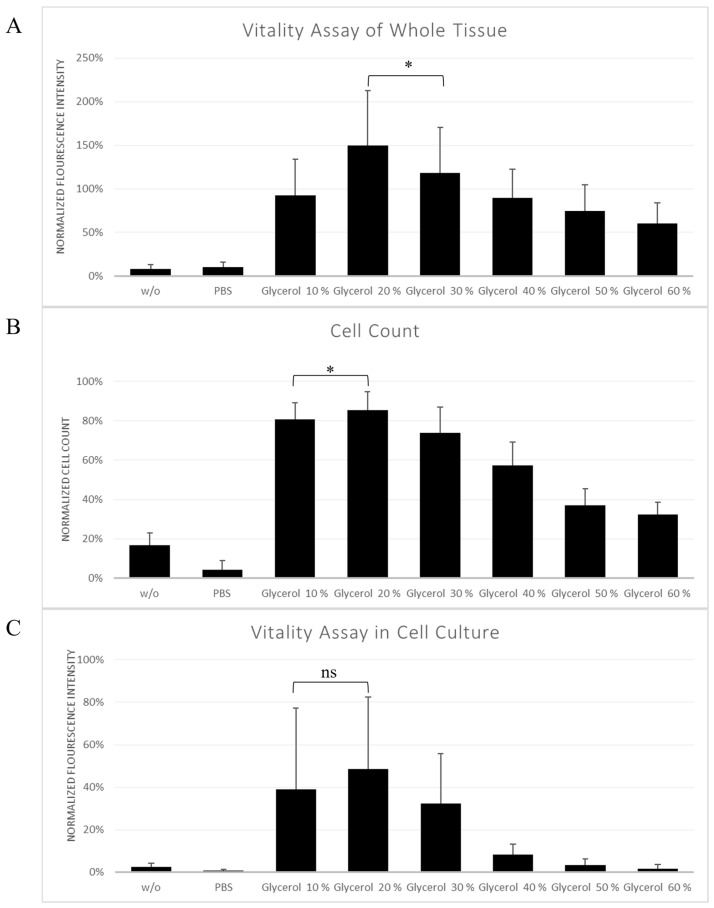
(**A**): Vitality assay of whole tissue. Fluorescence intensities, normalized to the corresponding fresh control. (**B**): Live/dead ratio of stromal vascular fraction cell counts, normalized to the corresponding fresh control. (**C**): Vitality assay after short-term cell culture, normalized to the corresponding fresh control. Mean values and standard deviations are shown. * = *p*-value < 0.05. ns: No significant differences.

As illustrated in Figure 1A, all groups cryopreserved in the presence of glycerol exhibited a higher fluorescence intensity compared with samples frozen without cryoprotectant (*w*/*o*) and with PBS alone. Among the glycerol treated groups, the 20% concentration demonstrated the highest fluorescence intensity and was significantly superior to the second highest fluorescence intensity found in 30% glycerol concentration (*p* < 0.05). Effect size analysis confirmed a large positive effect of 20% glycerol compared with *w*/*o* and all other glycerol-treated groups (Cohen’s d > 1.16 against glycerol 30% group).

### 3.2. Cell Count and Live/Dead Ratio of Stromal Vascular Fraction Cells

In the control group, a mean of 435,714.3 ± 76,877.9 surviving cells and a live/dead ratio of 90.93% ± 5.74% was measured. As illustrated in Figure 1B, cryopreservation with glycerol increased the number of surviving stromal vascular fraction cells compared with *w*/*o* and PBS-only groups (*p* < 0.05). The live/dead ratio of glycerol 20% samples remained significantly higher than that of the second highest live/dead ratio found in 10% glycerol concentration (*p* < 0.05).

Within the glycerol-treated groups, concentrations between 10 and 30 percent showed the highest surviving cell counts and live/dead ratios. The maximal preservation of surviving cells was observed at 20% percent glycerol. In contrast, glycerol concentrations of 40 percent and higher were associated with a decline in surviving cell yield and live/dead ratios compared with intermediate concentrations. Effect size analysis demonstrated large effects for glycerol in 20% concentration when compared with all other groups (d = 1.24 to 6.94).

### 3.3. Vitality Assay in Cell Culture

As shown in Figure 1C, glycerol-treated groups exhibited higher fluorescence intensities after 24 h of cell culture compared with *w*/*o* and PBS-only groups. All glycerol concentration demonstrated lower metabolic activity than fresh unfrozen control cells.

The highest post-thawing viability was observed at 20% glycerol, whereas concentrations of 40 percent and above resulted in reduced fluorescence intensities compared with intermediate concentrations. Samples frozen without cryoprotectant showed the lowest metabolic activity.

Effect size analysis confirmed large effects for glycerol 20% concentration compared with *w*/*o* and PBS-only samples (d = 1.68 and 0.86), while the effects were lower when compared to 10 and 30% glycerol groups (d = 0.40 and 0.79).

### 3.4. Effect Size Analysis (Cohen’s d)

As can be seen in Table 2 across all three assays, a glycerol concentration of 20% consistently yielded large effect sizes compared with cryopreservation without cryoprotectant and PBS-only controls, as well as against all other glycerol-preserved groups except for the vitality in cell culture for 10 and 30% glycerol.

In Figure 1B, the PBS group shows a lower living cell fraction compared to the *w*/*o* group.

### 3.5. Large-Volume Cryopreservation in Clinical Cryobags

**Figure 2:**
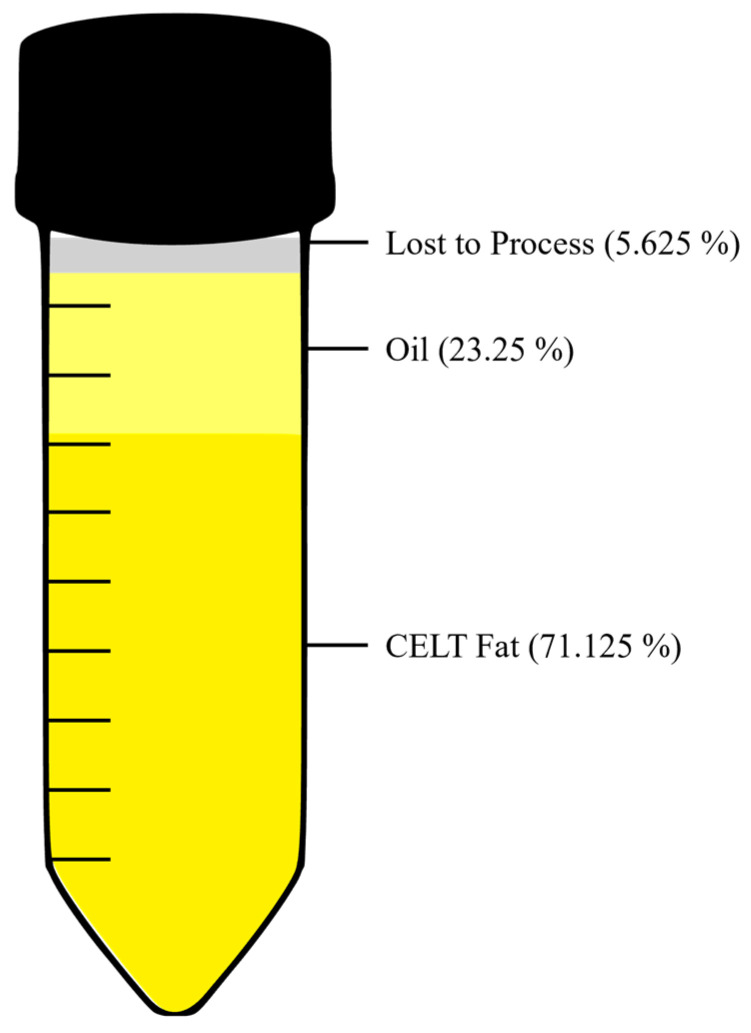
Distribution of components after thawing large-volume samples. The displayed stratification represents the actual phase separation after centrifugation of the sample in a centrifuge tube (Falcon), following initial freezing in a tissue bag. The schematic illustration depicts the final composition of material collected in the Falcon tube after processing, including oil, viable CELT fat, and material lost during the freezing process mainly due to adherence to the tissue bag. Values are presented as mean, (*n* = 4).

To assess large-volume cryopreservation under clinically applicable conditions, the same experimental assays applied to small-volume samples were performed on lipoaspirate cryopreserved in large-volume bags (Figure 2).

### 3.6. Vitality Assay of Whole Tissue in Large Volumes

**Figure 3:**
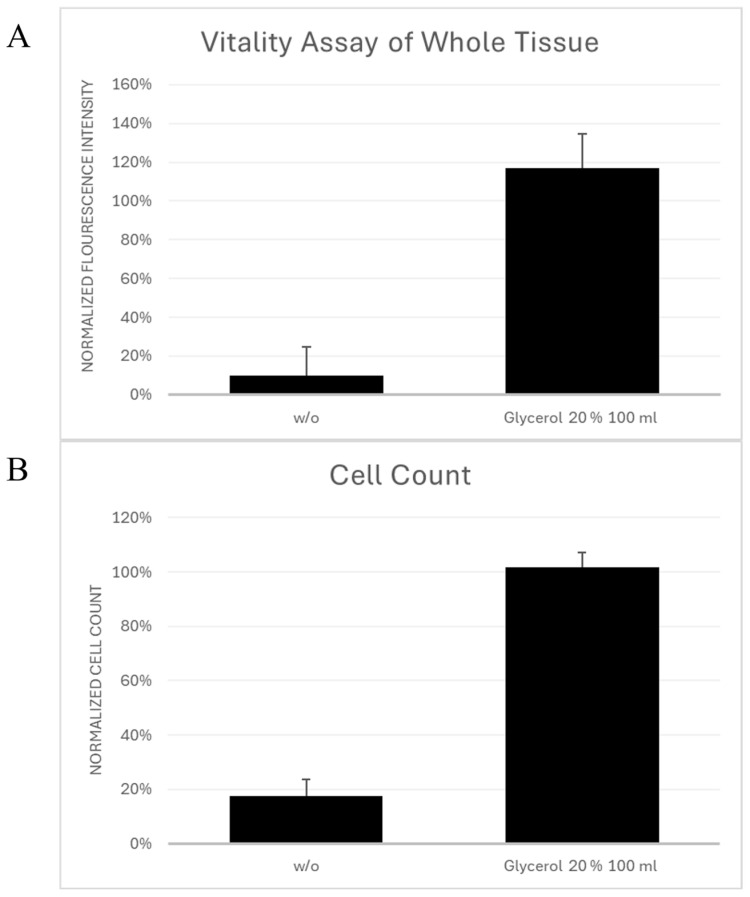
(**A**): Vitality assay of whole tissue normalized to the small-volume glycerol 20% 0.7 mL samples (**B**): Live/dead ratio of stromal vascular fraction cell counts normalized to the small-volume glycerol 20% 0.7 mL samples. Mean values and standard deviations are shown.

As shown in Figure 3A, fluorescence intensities of cryobag-preserved samples did not differ from those obtained in corresponding small-volume samples treated with 20% glycerol. Cryobag samples exhibited higher fluorescence intensities than samples cryopreserved without cryoprotectant. According to Cohen’s d seen in Table 3, the effect between both volumes was negligible.

### 3.7. Cell Count and Live/Dead Ratio

As illustrated in Figure 3B, the number of viable stromal vascular fraction cells isolated from cryobag-preserved samples was comparable to those obtained from small-volume glycerol treated samples. The live/dead ratio did not differ between cryobag- and vial-based cryopreservation. Compared with the *w*/*o* group, cryobag samples showed higher live/dead ratios. According to Cohen’s d seen in Table 3, the effect between both volumes was small.

## 4. Discussion

The present study demonstrates that human lipoaspirate can be successfully cryopreserved using a GMP-compatible workflow based on glycerol as a cryoprotective agent and large-volume cryobags. Our results indicate that glycerol concentrations between 10 and 30 percent provide effective cryoprotection, with an optimal performance observed at 20 percent glycerol, significantly better-performing than the second-best-performing glycerol concentrations in the viability assay and the live/dead ratio. Importantly, cryopreservation in large-volume bags did not result in inferior tissue quality compared to small laboratory samples, supporting the feasibility of clinical-scale fat banking.

Both the *w*/*o* and PBS groups demonstrated that, in the absence of sufficient cryoprotective agents, cell survival is severely limited. Moreover, the worsening of the live/dead ratio in the PBS-only group indicates that the beneficial effect observed with the glycerol-based mixture is not attributable to PBS. Rather PBS alone may even exert a detrimental effect during freezing, potentially due to the formation of damaging ice crystals.

In contrast to isolated cell populations, lipoaspirate represents a complex tissue composed of adipocytes, stromal vascular fraction cells, extracellular matrix and microvascular networks [39,40,41]. Preservation of this composite structure is essential for maintaining both volumetric stability and regenerative potential after transplantation. Our findings confirm that glycerol can protect tissue vitality and SVF cell survival during freezing and thawing while avoiding the cytotoxic risks associated with DMSO.

The choice of cryoprotective agent is of relevance in aesthetic and reconstructive surgery. DMSO, although widely used in experimental cryobiology, exhibits well-documented dose-dependent cytotoxicity and can induce inflammatory reactions after transplantation [42,43,44]. These effects are especially undesirable in oncologic patients, in chronically inflamed wounds and in repeated applications as in joint treatments. In this context, glycerol offers a biologically more favorable alternative. Its lower toxicity profile and long-standing clinical use make it particularly attractive for clinical fat banking [33,34,45].

Although no direct head-to-head comparison with DMSO was performed in the present study, previously published data from our group allows an indirect contextualization of glycerol’s performance relative to DMSO (Schimanski et al., 2025) [36]. In that earlier investigation, DMSO-based cryopreservation achieved satisfactory results in the resazurin assay, and the cell count of SVF cells after thawing is associated with cytotoxic effects at room temperature. Nevertheless, the viability rates and SCF cell survival observed in the current study with glycerol are favorable to those previously obtained with DMSO while offering a more favorable biological safety profile.

An important aspect of our study is the systematic evaluation of glycerol concentration. Other studies suggested beneficial results for cryopreservation with glycerol [33,34]. Our data demonstrate that concentrations between 10 and 30 percent provide robust cryoprotection, whereas higher concentrations lead to a marked decline in tissue survival. The superior performance observed at 20% glycerol suggests that this concentration offers an optimal balance between cryoprotection and osmotic tolerance.

It should be noted that cell counting using trypan blue only distinguishes live from dead cells at the time of counting and does not provide information on the subsequent functional capacity of the cells. To assess whether cells remain metabolically active and able to adhere and survive over time, a culture-based viability assay was performed. The viability results from the cell culture provides a functional survival assay of post-thaw cell survival at the tissue level. Unlike single-cell normalized measurements, it reflects the viability of all SVF cells able to adhere and maintain metabolic activity from a defined starting volume of CELT fat. Thus, differences in fluorescence between groups represent biologically relevant variations in functional survival rather than artifacts of seeding density. Therefore, we were able to show that even after cryopreservation the surviving cells were still functional for optimal concentrations of glycerol.

A key strength of our protocol is the use of large-volume cryobags. In the future, for patient usage, sterile bags are mandatory to fulfill GMP requirements. These bags allow closed-system processing directly in the operating room, enabling the immediate transfer of freshly harvested fat into a sterile cryopreservation environment. This approach minimizes the risk of microbial contamination and ensures full regulatory compliance.

Importantly, we observed no disadvantage of large-volume cryopreservation when directly compared to small-sample freezing. This finding has major clinical implications, as it enables the storage of clinically relevant fat volumes in a single or limited number of containers. A notable finding in our study was the release of free lipids following thawing of large-volume samples. We hypothesize that free lipids release also occurs in small volumes, but it is more difficult to detect as it constitutes only a small amount. This phenomenon reflects the rupture of mature adipocytes during the freezing and thawing process [22,46]. At first glance, this may appear as a limitation of the technique. However, we interpret this finding as a potential advantage rather than a drawback. The released oil can be efficiently removed by centrifugation according to established protocols such as the CELT protocol, resulting in a concentrated tissue fraction enriched in stromal and progenitor cells.

This selective loss of fragile mature adipocytes may lead to a relative enrichment of regenerative cell populations per volume unit. Such a shift in tissue composition could be particularly beneficial in regenerative indications, including chronic wound treatment, joint therapy and soft tissue regeneration after radiation or oncologic surgery. In these settings, the regenerative and immunomodulatory properties of the stromal vascular fraction are likely more relevant than pure volumetric augmentation [38,47,48]. Thus, the observed tissue fractionation may represent a biological optimization rather than a technical failure.

The concept of off-the-shelf autologous fat grafting enabled by cryopreservation has far-reaching clinical implications. In breast reconstruction after cancer surgery, repeated fat grafting sessions are often required, and repeated harvesting may be undesirable or impossible [49,50,51]. In joint therapies, repeated intraarticular applications are frequently performed with limited duration of effect [52,53,54]. In paraplegic patients, prophylactic fat grafting to the gluteal and sacral regions may play a critical role in pressure ulcer prevention [55,56,57].

In all these scenarios, the availability of banked autologous fat offers a unique therapeutic advantage. It reduces surgical burden, improves patient comfort and allows timely intervention without repeated donor site trauma. For many patients with limited alternatives, this strategy may represent a last therapeutic option and significantly improve quality of life.

Despite these promising results, several limitations of the present study must be acknowledged. Most importantly, while the presented cryopreservation protocol is designed to be compatible with GMP requirements, it does not by itself constitute a fully GMP-compliant process. Critical quality attributes such as identity, sterility, purity, and potency, as well as appropriate monitoring and documentation procedures, must be established and validated separately in accordance with regulatory standards. In addition, all materials used, including glycerol as a cryoprotective agent, must be certified and approved for clinical use prior to application in patients. The presented cryopreservation approach is, in principle, suitable for implementation within a GMP framework, if all required regulatory conditions are fulfilled.

Furthermore, long-term storage effects were not evaluated. While previous studies suggest that storage up to three months at −80 °C is not associated with significant deterioration, longer storage periods may require liquid nitrogen-based preservation [45,58,59]. Sterile cryobags available on the market are compatible with both storage modalities, allowing future optimization depending on intended storage duration.

Another limitation is the lack of in vivo data. Although our in vitro analyses demonstrate preserved tissue quality, the ultimate clinical performance of cryopreserved fat depends on graft survival, integration and regenerative efficacy after transplantation. These aspects should be addressed in future studies focusing on long-term storage and clinical outcome evaluation.

In some glycerol-treated groups, the measured metabolic activity immediately post-thaw exceeded that of the unfrozen control. This likely reflects a transient increase in cell metabolism upon recovery from cryostorage. While this limits direct comparison of absolute pre- versus post-thaw viability, relative differences between cryopreserved groups remain valid, and functional survival was further assessed using complementary assays.

In addition, the exploration of novel cryoprotectants and additive compounds represents an important future research direction.

Cryopreservation in liquid nitrogen (−196 °C) remains a gold standard for the long-term storage of cells and tissues due to an almost complete attenuation of molecular movement and cellular degeneration [60,61]. In contrast, at −80 °C certain physical and chemical processes remain active within preserved tissues and cells, although mitigated. With standardized protocols and the use of suitable cryopreservation agents, storage at −80 °C can be a more practical alternative, especially for short- and medium-term storage [21]. Moreover, this kind of cryopreservation offers logistic, safety and technical benefits due to its more efficient implementation and GMP integrity.

Finally, we emphasize the importance of quality control prior to clinical application. Independent of the cryopreservation protocol, post-thaw evaluation of tissue quality should be considered mandatory. Assessing viability, tissue integrity and sterility status before transplantation ensures optimal patient safety and therapeutic efficacy.

## 5. Conclusions

In the present study, we established a GMP-compatible cryopreservation protocol for human lipoaspirate based on glycerol as a cryoprotective agent and identified the optimal concentration for preservation of tissue quality to be 20% (*v*/*v*). Our results demonstrate that glycerol effectively protects adipose tissue during freezing and thawing and enables clinically relevant large-volume cryopreservation.

The identification of an optimal glycerol concentration provides an important step toward standardized fat banking protocols suitable for routine clinical application. The feasibility of large-volume storage under sterile, closed-system conditions supports the concept of off-the-shelf autologous fat grafting for patients requiring repeated reconstructive and regenerative interventions. Although long-term storage effects were not addressed in the present study, our findings establish a robust methodological basis for future investigations focusing on extended storage duration, clinical graft performance and functional regenerative outcomes. Glycerol-based cryopreservation in combination with the Cell-Enriched Lipotransfer (CELT) protocol for lipoaspirate processing represents a biologically rational and potentially safe strategy to enhance the long-term availability of viable adipose graft material. The prior removal of excess tumescent fluid, blood components, and free lipid fractions through optimized processing improves tissue purity and may reduce cryo-induced cellular stress. Glycerol acts as a penetrating cryoprotective agent, mitigating intracellular ice crystal formation, osmotic imbalance, and membrane destabilization during freezing and thawing. When applied to adipose tissue that has been processed under conditions preserving extracellular matrix integrity and stromal vascular niche viability and survival, this approach may contribute to improved post-thaw cell survival, maintenance of adipose-derived stromal/stem cell (ASC) functionality, and sustained regenerative capacity. Collectively, the combination of CELT-based tissue optimization and glycerol-mediated cryoprotection may facilitate repeated autologous applications, particularly in patients with limited donor-site availability or those requiring staged or recurrent regenerative treatments.

## Figures and Tables

**Table 2:** Cohen’s d effect sizes refer to the comparison of each experimental group with the 20% glycerol group across three experimental assays.

	*w/o*	PBS	Glycerol 10 %	Glycerol 30%	Glycerol 40%	Glycerol 50%	Glycerol 60%
Vitality assay of Whole Tissue	2.31	2.34	1.44	1.16	1.78	1.90	2.08
Cell Count	6.30	6.94	1.24	1.61	2.16	3.72	4.14
Vitality assay of Cell Culture	1.68	0.86	0.40	0.79	1.10	1.23	1.29

Color coding represents the magnitude of the effect size according to Cohen et al. [38]: Yellow: 0.2 ≤ d < 0.5 (small effect); Light green: 0.5 ≤ d < 0.8 (intermediate effect); Dark green: d ≥ 0.8 (large effect).

**Table 3:** Cohen’s d effect sizes refer to the comparison of each experimental group with the 0.7 mL 20% glycerol group across two experimental assays.

	*w/o*	Glycerol 20 % 100 mL
Vitality assay of Whole Tissue	1.58	0.06
Cell Count	6.66	0.30

Color coding represents the magnitude of the effect size according to Cohen et al. [38]: Grey: d < 0.2 (negligible effect); Yellow: 0.2 ≤ d < 0.5 (small effect); Dark green: d ≥ 0.8 (large effect).

## Data Availability

The original contributions presented in this study are included in the article. Further inquiries can be directed to the corresponding authors.
